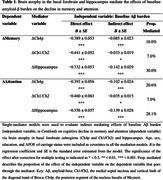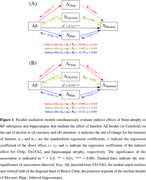# Association of basal forebrain atrophy with cognitive decline in early Alzheimer’s disease

**DOI:** 10.1002/alz.091113

**Published:** 2025-01-09

**Authors:** Ying Xia, Vincent Dore, Jurgen Fripp, Pierrick Bourgeat, Simon M. Laws, Christopher J Fowler, Stephanie R Rainey‐Smith, Ralph N Martins, Christopher C. Rowe, Colin L Masters, Elizabeth J Coulson, Paul Maruff

**Affiliations:** ^1^ CSIRO Health and Biosecurity, Australian E‐Health Research Centre, Brisbane, QLD Australia; ^2^ Austin Health, Melbourne, VIC Australia; ^3^ CSIRO Health and Biosecurity, Australian E‐Health Research Centre, Parkville, VIC Australia; ^4^ CSIRO, Brisbane, QLD Australia; ^5^ Collaborative Genomics and Translation Group, School of Medical and Health Sciences, Edith Cowan University, Joondalup, Western Australia Australia; ^6^ Curtin Medical School, Curtin University, Bentley, Western Australia Australia; ^7^ Centre for Precision Health, Edith Cowan University, Joondalup, Western Australia Australia; ^8^ The Florey Institute of Neuroscience and Mental Health, The University of Melbourne, Parkville, VIC Australia; ^9^ School of Psychological Science, University of Western Australia, Crawley, Western Australia Australia; ^10^ Australian Alzheimer's Research Foundation, Nedlands, Western Australia Australia; ^11^ School of Medical and Health Sciences, Edith Cowan University, Joondalup, Western Australia Australia; ^12^ Centre for Healthy Ageing, Murdoch University, Murdoch, Western Australia Australia; ^13^ Alzheimer's Research Australia, Perth, Western Australia Australia; ^14^ Edith Cowan University, Perth, Western Australia Australia; ^15^ Department of Biomedical Sciences, Macquarie University, Macquarie Park, NSW Australia; ^16^ Queensland Brain Institute, The University of Queensland, Brisbane, QLD Australia; ^17^ School of Biomedical Sciences, The University of Queensland, Brisbane, QLD Australia; ^18^ Cogstate Ltd., Melbourne, VIC Australia

## Abstract

**Background:**

In early Alzheimer’s disease (AD), amyloid‐β (Aβ) accumulation is associated with volume loss in the basal forebrain (BF) and cognitive decline. However, the extent to which Aβ‐related BF atrophy manifests as cognitive decline is not understood. This study sought to characterize the relationships between Aβ burden, BF atrophy, and the decline in memory and attention in older individuals without dementia.

**Method:**

The 476 older adults (72.6 ± 5.9 years old, 55.0% female) from the Australian Imaging, Biomarker and Lifestyle (AIBL) study were included, who underwent Aβ‐PET imaging at baseline and repeated MRI and cognitive assessments over a period up to 13 years. At baseline, participants were classified based on their clinical dementia stage and Aβ status, which were cognitively unimpaired (CU) Aβ−, CU Aβ+, and mild cognitive impairment (MCI) Aβ+. Linear mixed‐effects models assessed atrophy in BF subregions (Ch4p – posterior segment of the nucleus basalis of Meynert; Ch1/Ch2 ‐ medial septal nucleus and vertical limb of the diagonal band of Broca) and hippocampus as well as changes in their AIBL memory and attention composite scores. Associations between baseline Aβ burden, brain atrophy, and cognitive decline were evaluated and explored using mediation analyses.

**Result:**

Compared to the CU Aβ− group, CU Aβ+ and MCI Aβ+ adults both showed faster decline in BF and hippocampal volumes as well as in memory and attention (all *p* < 0.001). While rates of atrophy across all regions correlated with cognitive decline, baseline Ch4p volume was associated moderately with rates of memory and attention decline (*p* < 0.001, η^2^ = 0.046 and 0.023). Furthermore, single‐mediator models demonstrated that rates of atrophy in all regions of interest significantly influenced the effects of Aβ burden on memory and attention decline (Table 1). When considering all three mediators simultaneously, hippocampal atrophy primarily mediated the effects of Aβ burden on memory decline (Figure 1A), whereas Ch4p and hippocampal atrophy play unique mediating roles in Aβ‐related attention decline (Figure 1B).

**Conclusion:**

These findings underscore the important role of early volume loss in the BF, especially in Ch4p, in the complex pathway linking Aβ to cognitive impairment in early AD.